# Late Breaking Biological Sciences Session

**DOI:** 10.1093/geroni/igad104.0325

**Published:** 2023-12-21

**Authors:** Blanka Rogina

## Abstract

This is a key element specific to the Biological Sciences Section within the Gerontological Society of America annual scientific meeting that allows for very new and unpublished findings to be presented. Due to the rapid advancement of our knowledge, four speakers in this symposium are selected from the submitted late breaking Abstracts. Special emphasis is given to selecting early career faculty, postdoctoral fellows and graduate students.

